# The Relationship Between Workplace Ostracism and Organizational Dehumanization: The Role of Need to Belong and its Outcomes

**DOI:** 10.5334/pb.1215

**Published:** 2023-11-08

**Authors:** Noémie Brison, Gaëtane Caesens

**Affiliations:** 1 Psychological Sciences Research Institute, Université catholique de Louvain, Louvain-la-Neuve, Belgium

**Keywords:** workplace ostracism, organizational dehumanization, depression, job satisfaction, turnover intentions, loyalty behaviors

## Abstract

This research investigates whether experiencing workplace ostracism is positively related to employees’ perceptions of organizational dehumanization, and examines one underlying mechanism of this relationship (i.e., thwarted need to belong), as well as its consequences for both employees and organizations. First, a cross-sectional study (*N* = 256) highlighted that workplace ostracism positively relates to organizational dehumanization which, in turn, is related to employees’ well-being (i.e., increased depression), attitudes (i.e., decreased job satisfaction) and behaviors toward the organization (i.e., increased turnover intentions, decreased loyalty behaviors toward the organization). Second, an experimental study manipulating workplace ostracism using vignettes (*N* = 199) showed that workplace ostracism has a positive impact on organizational dehumanization, which subsequently relates to employees’ decreased job satisfaction, increased turnover intentions, and decreased loyalty behaviors. Finally, a third cross-sectional study (*N* = 423) revealed that employees’ thwarted need to belong mediates the relationship between workplace ostracism and organizational dehumanization, which is ultimately associated with employees’ increased depression, decreased job satisfaction, increased turnover intentions, and decreased loyalty behaviors. Theoretical contributions, directions for future research and practical implications are discussed.

Workplace ostracism, referring to any situation where “an individual or group omits to take actions that engage another organizational member when it is socially appropriate to do so” (Robinson et al., 2013, p.206), has attracted scholarly attention across the past years (e.g., Ferris et al., 2008; Howard et al., 2020). Indeed, as illustrated by recent meta-analytic findings indicating that 45% of employees experienced ostracism at work (Dhanani et al., 2021), exclusionary phenomena in organizations are commonplace. Such scholarly interest is also underlined by the observation that workplace ostracism may be more detrimental to employees’ well-being, attitudes and behaviors than other forms of mistreatment (O’Reilly et al., 2015).

Although workplace ostracism is thought to occur because organizations often fail to detect and punish this covert and ambiguous mistreatment (e.g., Robinson et al., 2013), prior research has mainly considered the organization as an innocent bystander of these exclusionary phenomena. Yet, some studies suggest that victims of workplace ostracism might hold the organization partly responsible for this negative treatment received by its members (e.g., Howard et al., 2020; Paşamehmetoğlu et al., 2022). For instance, recently, workplace ostracism was found to be negatively related to organizational trust (e.g., Paşamehmetoğlu et al., 2022). Additionally, prior studies reported that employees generalize the (positive or negative) treatment they received from their supervisor or their coworkers to the entire organization (e.g., Caesens et al., 2019; Hayton et al., 2012; Stinglhamber et al., 2015).

As impaired relationships between employees and their organization are detrimental for both parties, there is a need to thoroughly understand how workplace ostracism might lead to damaged perceptions of the overall organization and their subsequent negative consequences for employees and organizations (e.g., Paşamehmetoğlu et al., 2022; Wu et al., 2016). In this research, we thus propose to examine organizational dehumanization (i.e., “the experience of an employee who feels objectified by his or her organization, denied personal subjectivity, and made to feel like a tool or instrument for the organization’s ends”; Bell & Khoury, 2011, p.170) as an underlying mechanism explaining the consequences of workplace ostracism.

More precisely, this research has three main objectives. First, it examines for the first time the positive relationship between workplace ostracism and organizational dehumanization. Second, it investigates whether this relationship is linked to the three main categories of outcomes of organizational dehumanization (Brison et al., 2022), that is employees’ well-being (i.e., increased depression), attitudes (i.e., decreased job satisfaction), and behaviors (i.e., increased turnover intentions, decreased loyalty behaviors). Finally, it seeks to identify one underlying mechanism (i.e., thwarted need to belong) which might explain the positive relationship between workplace ostracism and organizational dehumanization. The theoretical model is presented in Figure 1.

**Figure 1:**
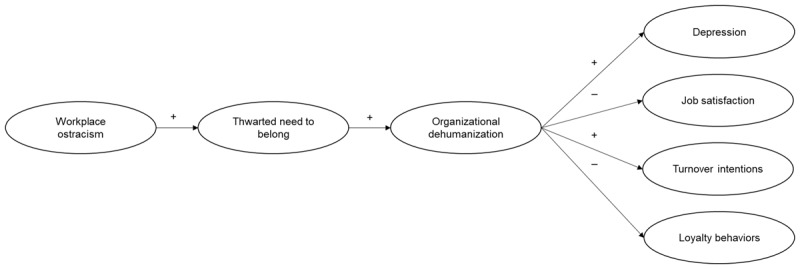
Hypothesized Model. *Note*: Hypothesized mediation effects are expected to be at least partial.

While pursuing these objectives, this research provides several contributions to the literature on workplace ostracism and organizational dehumanization. First, by highlighting that workplace ostracism relates to victims’ poorer well-being, attitudes, and behaviors through organizational dehumanization, this research might help to bring the fragmented workplace ostracism literature into an overarching model (Howard et al., 2020). Indeed, prior work on workplace ostracism mostly relied on frameworks that only explained certain subsets of its outcomes, resulting in a poor holistic understanding of this phenomenon (Howard et al., 2020). Second, while it is established that organizational dehumanization arises from abusive supervision (Caesens et al., 2019), this research might show that an indirect and ambiguous mistreatment from both supervisors and coworkers (i.e., workplace ostracism) relates to organizational dehumanization perceptions (Brison et al., 2022). Overall, by exploring the workplace ostracism-organizational dehumanization relationship, its underlying mechanism and outcomes, this research might provide a more comprehensive understanding of the organizational dehumanization construct and its nomological network. Finally, at the practical level, identifying drivers of organizational dehumanization might have implications for practitioners as it might help them better understand the role of ostracism in the development of the negative organizational phenomenon that is organizational dehumanization.

## Workplace Ostracism

Workplace ostracism refers to employees’ perceptions that they have been ignored or excluded at work (Ferris et al., 2008). It is typically described as an interpersonal mistreatment perpetrated by organizational members (i.e., supervisor, coworkers) that is conceptually distinct from other interpersonal mistreatments such as workplace incivility (Ferris et al., 2017). Specifically, workplace ostracism implies an omission of act (i.e., absence of attention) whereas other forms of mistreatment involve the commission of acts (i.e., negative attention) (Ferris et al., 2017). As such, workplace ostracism is distinct from other constructs such as professional isolation, which refers to employees’ beliefs that they are disconnected from others at work due to practical constraints (e.g., remote working) (Peng et al., 2023).

As it indicates to employees that they are not even worth receiving negative attention, workplace ostracism is an experience whereby the existence of employees as human beings is fully denied (O’Reilly et al., 2015). Consistent with this, workplace ostracism is thought to threaten employees’ fundamental needs (Robinson et al., 2013), which in turn is associated with poorer employees’ well-being (e.g., increased emotional exhaustion and depression), more negative work-related attitudes (e.g., decreased job satisfaction) and behaviors (e.g., decreased performance) (Howard et al., 2020). Additionally, recent studies suggested that workplace ostracism might be related to negative perceptions of the organization, such as by reducing employees’ organizational identification (e.g., Wu et al., 2016), affective organizational commitment (e.g., Howard et al., 2020) or organizational trust (e.g., Paşamehmetoğlu et al., 2022). Scholars further argued that workplace ostracism might signal employees’ decreased worth to the organization (Jahanzeb et al., 2018). In line with this, in this research, we propose that workplace ostracism might trigger organizational dehumanization perceptions.

## Organizational Dehumanization

The concept of organizational dehumanization is rooted in the social psychology literature, which defines dehumanization as the denial of one’s human characteristics (Haslam, 2006). Drawing on Haslam’s dual model of dehumanization (2006), scholars distinguish animalistic dehumanization that arises when individuals are denied features that differentiate them from animals (i.e., civility, refinement, morality, rationality, maturity) and mechanistic dehumanization that emerges when individuals are denied attributes that distinguish them from objects or machines (i.e., emotionality, interpersonal warmth, cognitive openness, agency, depth). Since dehumanization occurs within organizational contexts, especially its mechanistic form, scholarly attention has shifted to organizational dehumanization, which refers to employees’ perceptions to be objectified, denied subjectivity, and treated like interchangeable tools by the organization to achieve its goals (Bell & Khoury, 2011). Scholars argued that these perceptions are likely to arise in today’s workplace characterized, for instance, by injunctions to increase profits, bureaucracy, technological breakthroughs, and indecent work conditions (Brison et al., 2022). As such, organizational dehumanization is typically described as a harmful phenomenon that entails detrimental consequences for both employees (e.g., increased emotional exhaustion, decreased job satisfaction; Lagios et al., 2022; Stinglhamber et al., 2021) and organizations (e.g., increased organizational deviance, turnover intentions, decreased safety participation; Caesens & Brison, 2023; Bell & Khoury, 2016; Stinglhamber et al., 2023).

Because of the detrimental nature of organizational dehumanization (e.g., Brison et al., 2022), scholarly interest has been devoted to the identification of its predictors. So far, prior work examined factors related to organizational values and practices (e.g., perceived organizational support; Caesens et al., 2017), to the work environment (e.g., flex-desks; Taskin et al., 2019), and to the job itself (e.g., job autonomy; Demoulin et al., 2020). Additionally, several scholars suggested that interpersonal mistreatment from organizational members, such as the supervisor, coworkers or workgroup, may be a driver of organizational dehumanization (e.g., Brison et al., 2022). Accordingly, based on attribution theory (Kelley, 1973), scholars suggested that employees are likely to seek causal attributions for the way they are treated by significant organizational members (e.g., supervisors and coworkers), resulting in responsibility and blame attributions (Bowling & Beehr, 2006). Specifically, following interpersonal mistreatment (e.g., workplace ostracism), “victims can easily blame the organization for its climate and for the existence of certain role stressors” (Bowling & Beehr, 2006, p.1000). Similarly, Paşamehmetoğlu et al. (2022) claimed that “employees might blame the organization for allowing or being complicit with the development of ostracism in the workplace” (p.124).

While the “immediate managers instantiate the organization” (Ashforth & Rogers, 2012, p.35), employees often have multiple coworkers with whom they usually interact more frequently (Seers, 1989). Because supervisors and coworkers are important members of the organization, employees perceive the treatment they receive from their supervisor or coworkers as illustrative of the treatment they receive from their entire organization (e.g., Ashforth & Rogers, 2012; Shoss et al., 2013). Consistently, abusive supervision (Caesens et al., 2019) and leader-member exchange (Stinglhamber et al., 2021) were found to be positively and negatively associated with organizational dehumanization perceptions, respectively. Similarly, Väyrynen and Laari-Salmela (2018) claimed that mistreatments received by omission, such as workplace ostracism, “reflect a disregard for the essential humanness of employees and relate to the way that people are treated within the organization” (Väyrynen & Laari-Salmela, 2018, p.108), thereby fostering organizational dehumanization. Yet, this assumption has never been empirically tested.

Based on the above arguments, we therefore hypothesized the following:

Hypothesis 1: Workplace ostracism is positively related to organizational dehumanization.

## The Mediating Role of Organizational Dehumanization in the Workplace Ostracism-Outcomes Relationships

Numerous studies highlighted the deleterious consequences of workplace ostracism for both employees and organizations (e.g., Howard et al., 2020). Because it thwarts employees’ basic needs, especially the need to belong, workplace ostracism has been found to be linked to increased employees’ emotional exhaustion, lowered job satisfaction, and work engagement (e.g., Ferris et al., 2008; Howard et al., 2020). Moreover, past research indicated that workplace ostracism is positively related to employees’ turnover intentions and is negatively associated with employees’ in-role performance and organizational citizenship behaviors (e.g., Howard et al., 2020).

Similarly, scholars suggested that organizational dehumanization “is a negative experience that diminishes the individual and is therefore likely to motivate the individual to dissociate from the organization” (Bell & Khoury, 2011, p.184). As such, organizational dehumanization is argued to be associated with three main categories of outcomes: employees’ (1) well-being, (2) attitudes toward their work and organization, and (3) behaviors (Brison et al., 2022). In line with this, prior research reported that organizational dehumanization positively relates to employees’ emotional exhaustion (e.g., Stinglhamber et al., 2021) and somatic symptoms (e.g., Caesens et al., 2017). Furthermore, organizational dehumanization was found to negatively relate to employees’ job satisfaction (e.g., Lagios et al., 2022) and extra-role performance (e.g., Taskin et al., 2019), and to positively relate to their turnover intentions (e.g., Bell & Khoury, 2016).

Based on our first hypothesis and the above arguments, we thus posited that workplace ostracism is associated with organizational dehumanization perceptions which, in turn, are related to outcomes. Specifically, we focused on employees’ impaired well-being (i.e., increased depression), attitudes (i.e., decreased job satisfaction), and behaviors (i.e., increased turnover intentions, decreased loyalty behaviors). These outcomes were retained based on prior evidence suggesting that they represent their category (i.e., well-being, attitudes, behaviors) accurately. Specifically, the detrimental influence of mistreatment on victims’ well-being is most often conceptualized as mental health problems such as depressive symptoms (Ferreira et al., 2020). In addition, job satisfaction is considered as an important facet of employees’ attitudes toward their job (e.g., Eisenberger et al., 1997) and is a widely studied attitudinal outcome of workplace ostracism (Howard et al., 2020) and organizational dehumanization (Brison et al., 2022). Similarly, turnover intentions and loyalty behaviors (i.e., defending the organization and ensuring its good reputation to outsiders; Van Dyne et al., 1994) are key behavioral outcomes in organizational research, as they are indicative of the extent to which the organization might lose (Jaros, 1997) or attract (Van Hoye & Lievens, 2007) talented individuals, respectively.

Hypothesis 2: Organizational dehumanization (at least partially) mediates the relationship between workplace ostracism and employees’ (a) depression, (b) job satisfaction, (c) turnover intentions and (d) and loyalty behaviors toward the organization.

## The Mediating Role of Thwarted Need to Belong in the Workplace Ostracism-Organizational Dehumanization Relationship

On top of exploring whether workplace ostracism relates to negative outcomes via organizational dehumanization, this research also aims at identifying one underlying mechanism of the workplace ostracism-organizational dehumanization relationship. Specifically, scholars proposed that the reason why harmful interpersonal experiences relate to employees’ organizational dehumanization perceptions is because these experiences threaten victims’ fundamental needs (Baldissarri & Fourie, 2023). Therefore, we suggest that the frustration of employees’ need to belong explains the workplace ostracism-organizational dehumanization relationship. Indeed, some theoretical arguments and prior evidence support this assumption. On the one hand, workplace ostracism removes employees from the workgroup, thereby preventing them from building social connections (Ferris et al., 2008). Consequently, workplace ostracism might pose a threat to employees’ human desire for valuable interactions with others (Robinson et al., 2013). Since receiving social attention signals individuals that they belong to a group, the absence of attention such as workplace ostracism constitutes a threat to employees’ sense of belonging (O’Reilly et al., 2015). In line with this, prior research indicated that workplace ostracism positively relates to employees’ thwarted need to belong (e.g., Ferris et al., 2008; Howard et al., 2020).

On the other hand, since fundamental psychological needs are inextricably linked to humanness, prior scholars argued that “social targets will perceive a maltreatment as dehumanizing when their fundamental needs are undermined” (Demoulin et al., 2020, p.4), including their need to belong (Demoulin et al., 2020). Along similar lines, employees whose need to belong is thwarted are argued to feel dehumanized by the organization because their organization has neglected their most basic human needs (Baldissarri & Fourie, 2023). Corroborating this, empirical findings showed that employees’ thwarted need to belong is positively related to employees’ organizational dehumanization perceptions (Demoulin et al., 2020). Hence, we hypothesized the following:

Hypothesis 3: Employees’ thwarted need to belong (at least partially) mediates the positive relationship between workplace ostracism and organizational dehumanization.

## Overview of the Studies

Three studies, approved by the ethics committee of the authors’ institution, were conducted to explore the relationship between workplace ostracism and organizational dehumanization, one of its underlying mechanisms, and subsequent outcomes. A first cross-sectional field study investigates whether workplace ostracism positively relates to organizational dehumanization (H1), which in turn relates to employees’ well-being (i.e., increased depression), attitudes (i.e., decreased job satisfaction) and behaviors (i.e., increased turnover intentions, decreased loyalty behaviors) (H2a–d). Study 2, an experimental study where workplace ostracism was manipulated using vignettes, goes one step further by investigating the directionality of the relationship between workplace ostracism and organizational dehumanization. Specifically, it examines the relationship between workplace ostracism and organizational dehumanization (H1) and whether it subsequently relates to outcomes (i.e., job satisfaction, turnover intentions, loyalty behaviors) (H2b–d).^1^ Finally, Study 3 was a cross-sectional field study that replicates the findings of Study 1 and 2 and extends them by examining whether employees’ thwarted need to belong mediates the relationship between workplace ostracism and organizational dehumanization (H3) (see Figure 1).

## Study 1

### Method

#### Participants and Procedure

Participants who took part in this field study using online questionnaires were recruited via Prolific Academic and were paid £0.92 for their participation. To take part in the study, participants were required to have an approval rate on the platform of at least 95%, be native English speakers, and not be students nor self-employed. Additionally, since this study was conducted during the second main wave of the COVID-19 pandemic (i.e., March 2021), we added a criterion requiring participants to commute to work every day despite the pandemic. Indeed, since this research focuses on workplace ostracism, it was of primary importance to recruit participants working alongside coworkers and supervisors in person. Eighteen participants were excluded due to wrong answers to attentional questions, resulting in a final sample of 256 participants (*M*_age_ = 40.24, *SD* = 11.39; 44.5% women, 55.5% men) who had been working in their organization for an average of 8.42 years (*SD* = 7.41). Participants worked primarily in the health and social care domain (28.5%), in the field of retail and sales (16.4%), and in the engineering and manufacturing area (10.5%). Most participants held a bachelor’s degree (35.5%) and worked full-time (79.7%).

#### Measures

All items were assessed using a 7-point Likert scale ranging from “1” (strongly disagree) to “7” (strongly agree). Full measurement scales for all studies are provided in the online supplements.

**Workplace Ostracism (α = .96)** was assessed using the 10-item scale developed by Ferris et al. (2008) (e.g., “Others ignored me at work”). Prior studies reported good psychometric properties of this scale (e.g., Ferris et al., 2008), which is commonly used to assess workplace ostracism (e.g., Ferris et al., 2017; Howard et al., 2020).

**Organizational Dehumanization (α = .96)** was assessed with the 11-item scale developed by Caesens et al. (2017) (e.g., “My organization treats me as if I were an object”).

**Depression (α = .84)**. Participants’ levels of depression were measured with the 5-item scale from Bohannon et al. (2003) (e.g., “I felt depressed”).

**Job Satisfaction (α = .92)** was measured using the four items from Eisenberger et al. (1997) (e.g., “All in all, I›m very satisfied with my current job”).

**Turnover Intentions (α = .94)** were assessed using the three items from Jaros (1997) (e.g., “I intend to leave my organization in a near future”).

**Loyalty behaviors (α = .90)** were measured with four items from the loyalty subscale from Van Dyne et al. (1994). Following recommendations of Barnette (2000), two of the four items were adapted to get rid of double negatives (e.g., “I represent my organization favorably to outsiders”) in order to avoid reduced internal consistency.

**Control Variables**. We followed scholars’ recommendations to deal with sociodemographic control variables (Aguinis et al., 2017; Becker, 2005). Because controlling for sociodemographic variables that are uncorrelated with the dependent variables reduces power (Becker, 2005), only sociodemographic variables that were significantly associated with the dependent variables of the model (see Table 1) were included as control variables (Aguinis et al., 2017). All analyses were performed with and without these sociodemographic variables as control variables, and the findings were compared (Aguinis et al., 2017; Becker, 2005). As their inclusion in the analyses did not change the interpretation of the findings, the results reported here were free from any demographic variables for the sake of parsimony (Becker, 2005). Additionally, since abusive supervision is an interpersonal mistreatment that affects organizational dehumanization (Caesens et al., 2019), it was included as a theoretically relevant control variable. It was assessed with the five items from Mitchell and Ambrose (2007) using a 7-point Likert scale (e.g., “My supervisor ridicules me”; α = .94).

**Table 1:** Descriptive Statistics and Correlations among Variables for Study 1.


VARIABLE	*M*	*SD*	1	2	3	4	5	6	7	8	9	10	11	12	13	14	15	16

**1.** *Gender*	—	—	—															

**2.** *Age*	40.24	11.39	–.08	—														

**3.** *Organizational sector*	—	—	.15*	.07	—													

**4.** Type of contract	—	—	.23***	.07	.09	—												

**5.** Hierarchical status	—	—	.03	–.07	.07	.13*	—											

**6.** Organizational size	—	—	.04	–.01	.27***	.09	–.01	—										

**7.** Organizational tenure (years)	8.42	7.41	.03	.40***	.15*	.00	–21***	.10	—									

**8.** *Tenure–coworkers (years)*	5.04	4.41	.02	.34***	.03	.01	–.12	–.08	.64***	—								

**9.** *Tenure–supervisor (years)*	4.12	3.74	.03	.22***	–.10	.04	–.12*	–.20**	.40***	.64***	—							

**10. Abusive supervision**	1.78	1.15	.13*	–.15*	–.03	.04	.01	–.12	–.08	–.02	–.01	—						

**11.** Workplace ostracism	1.99	1.26	.04	–.10	.10	.04	.04	–.10	–.03	–.03	–.07	.61***	—					

**12.** Organizational dehumanization	4.33	1.58	.06	–.15**	.04	.02	.04	.12	.01	–.13*	–.15*	.33***	.37***	—				

**13.** Depression	3.19	1.50	.10	–.10	–.03	.08	–.01	–.03	–.07	–.02	.02	.39***	.38***	.34***	—			

**14.** Job satisfaction	4.62	1.55	.07	.08	.17**	.01	.01	.00	–.05	–.04	–.01	–.30***	–.28***	–.52***	–.38***	—		

**15.** Turnover intentions	3.58	1.90	.18**	–.20**	–.07	.04	–.04	–.04	–.05	–.03	–.02	.42***	.37***	.63***	.50***	–.68***	—	

**16.** Loyalty behaviors	4.25	1.41	–.01	.09	.05	–.06	–.11	–.10	–.01	.02	.06	–.12	–.18***	–.50***	–.22***	.72***	–.52***	—


*Note*: *N* = 256 (excepted for Tenure-coworkers *N* = 254, and for Tenure-supervisor *N* = 255). Gender was coded –1 = male and +1 = female. Organizational sector was coded –1 = private sector and +1 = public sector. Type of contract was coded 1 = full-time, 2 = 4/5 time, 3 = 3/4 time, and 4 = half-time. Hierarchical status was coded 1 = executive, 2 = supervisor management, 3 = middle manager, 4 = employee, and 5 = laborer. Organizational size was coded from 1 = less than 10 people to 9 = more than 10000 people. Abusive supervision is in bold because it was included as a control variable. The hypotheses were tested with and without the sociodemographic variables in italic as control variables, but these were not included in the final model for parsimony reasons. *p < .05. **p < .01. ***p < .001.

### Results

Descriptive statistics and correlations among variables are displayed in Table 1.

#### Measurement Model

Confirmatory factor analyses (CFA) were conducted to examine the distinctiveness of the seven constructs (i.e., workplace ostracism, abusive supervision, organizational dehumanization, depression, job satisfaction, turnover intentions, and loyalty behaviors) included in the model (Mplus 8.5, MLR estimator). Results indicated that the seven-factor model fitted the data well (χ^2^(798) = 1279.24; RMSEA = .05; SRMR = .04; CFI = .94; TLI = .94) and was superior to all more constrained models (see Table S1 in the online supplements).

#### Structural Model

**Figure 2:**
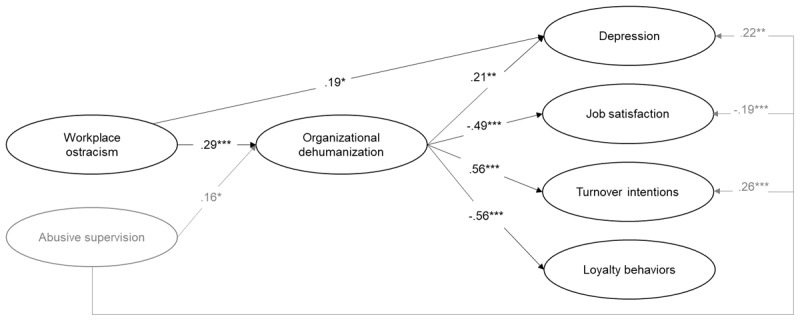
Standardized Coefficients for the Retained Structural Equation Model of Study 1. *Note*: *N* = 256. Abusive supervision was included as a control variable. All constructs represented in the figure are latent variables. * *p* < .05. ** *p* < .01. *** *p* < .001.

The hypotheses were tested using structural equation modeling (SEM). Specifically, we tested a model where workplace ostracism relates to the final outcomes (i.e., depression, job satisfaction, turnover intentions, loyalty behaviors), both directly and indirectly, through organizational dehumanization, while controlling for abusive supervision. Although the aforementioned model, including all possible direct paths between variables, displayed a satisfying fit with the data (χ^2^(798) = 1279.24; RMSEA = .05; SRMR = .04; CFI = .94; TLI = .94), alternative models were tested removing these direct paths one-by-one (see Table S2 in the online supplements). Results indicated that an alternative model keeping direct paths only between (1) workplace ostracism and depression as well as between (2) abusive supervision and depression, job satisfaction, and turnover intentions was equivalent to the model including all direct paths (χ^2^(802) = 1279.02; RMSEA = .05; SRMR = .05; CFI = .94; TLI = .94). For the sake of parsimony, this alternative model was retained as the final model (Figure 2).

Results indicated that, when controlling for abusive supervision, workplace ostracism was positively related to organizational dehumanization (γ = .29, *p* < .001). In turn, organizational dehumanization was positively related to depression (β =. 21, *p* < .01), negatively related to job satisfaction (β = –.49, *p* < .001), positively related to turnover intentions (β = .56, *p* < .001), and negatively related to loyalty behaviors (β = –.56, *p* < .001). Moreover, workplace ostracism was directly and positively linked to depression (γ = .19, *p* = .022). Latent bootstrap analyses (10000 bootstrap) also indicated that, when controlling for abusive supervision, the indirect effects of workplace ostracism on outcomes via organizational dehumanization were significant (depression = .061, 95% CI [.021, .122]; job satisfaction = –.141, 95% CI [–.226, –.069]; turnover intentions = .162, 95% CI [.080, .257]; loyalty behaviors = –.161, 95% CI [–.257, –.080]), supporting Hypotheses 1 and 2a–d. Overall, organizational dehumanization partially mediated the relationship between workplace ostracism and depression, while it fully mediated the relationships between workplace ostracism and job satisfaction, turnover intentions, and loyalty behaviors.

## Study 2

### Method

#### Participants and Design

Similar to Balliet and Ferris (2013), we used vignettes to manipulate workplace ostracism as they are the most appropriate method to overcome ethical dilemmas when examining causality between sensitive variables (Aguinis & Bradley, 2014). This experimental study was preregistered on Open Science Framework (https://osf.io/njr98). A power analysis was conducted to determine the appropriate sample size. Since the effect of workplace ostracism on organizational dehumanization has never been investigated experimentally before, we relied on an average effect size (*d* = .42; Richard et al., 2003). The analysis revealed that a minimal sample size of *N* = 196 was necessary for at least 90% power (α = .05). Anticipating the exclusion of participants failing the attentional question, 201 participants were recruited via Prolific Academic and were offered £0.59. They were eligible to participate if their approval rate on the platform was 90% or higher, were native English speakers, were not students nor self-employed, and if they did not take part in Study 1 nor in the pilot study conducted to pretest the vignettes. Two participants were excluded as they failed the attentional question. The final sample was composed of 199 participants (*M*_age_ = 41.62, *SD* = 10.78; 54.3% women, 44.7% men, 1% other).

Regarding the procedure, participants were randomly assigned to one of the two conditions (i.e., high versus low workplace ostracism). In both conditions, they were instructed to carefully read a short text (i.e., the description of an employee’s working day in a fictive company who experiences high versus low levels of workplace ostracism; see online supplements), and were invited to take the perspective of the employee of the vignette to rate the dependent variables. After that, they completed the manipulation check and a self-affirmation task to prevent any psychological damage, before being fully debriefed. As the vignettes were created for this study, they were first pretested in a pilot study. Fifty participants were recruited on Prolific Academic but one of them was excluded from the analyses due to a wrong answer to one attentional question. The final sample was composed of 49 participants (*M*_age_ = 38.37, *SD* = 9.57; 67.3% women, 32.7% men). After reading the vignette, they were asked to complete the workplace ostracism scale used in Study 1 (Ferris et al., 2008) as a manipulation check. Results showed that in the high workplace ostracism condition, participants reported higher levels of workplace ostracism (*M* = 6.53; *SD* = 0.68) as compared to the low workplace ostracism condition (*M* = 1.69; *SD* = 0.84), *t*(47) = 22.36, *p* < .001), supporting the effectiveness of the manipulation.

#### Measures

Organizational dehumanization (α = .96), job satisfaction (α = .97), turnover intentions (α = .98), and loyalty behaviors (α = .97) were measured using the same scales as in Study 1 (see the online supplements for the full scales).

**Manipulation Check**. At the end of the experiment, participants answered a multiple-choice question regarding the content of the vignettes (“Regarding the description of the day that you were asked to read at the beginning of the survey, which statement is true?”) and were provided with three response options. In the high workplace ostracism condition, the right response option was “Your colleagues did not pay attention to your suggestion during the meeting and they shared a coffee without you after the meeting”. In the low workplace ostracism condition, the right response option was “Your suggestion during the meeting raised the interest of your colleagues and you shared a coffee with them after the meeting” (for a full description, see online supplements). In each condition, most participants chose the right option (96% in the high workplace ostracism condition, *Z* = 9.25, *p* < .001; 99% participants in the low workplace ostracism condition, *Z* = 9.69, *p* < .001), highlighting that the manipulation was efficient. The analyses were conducted with and without participants who chose incorrect options. Because their exclusion did not change the interpretations of the findings, we reported the results from the full sample.

**Control Variables**. Again, we followed scholars’ (Aguinis et al., 2017; Becker, 2005) recommendations to deal with sociodemographic control variables. Specifically, since no sociodemographic variable significantly correlated with the dependent variables of the model (Table 2), none of them was included in the final model.

**Table 2:** Descriptive Statistics and Correlations among Variables for Study 2.


	*M*	*SD*	1	2	3	4	5	6	7

**1.** Gender	—	—	—						

**2.** Age	41.62	10.78	–.07	—					

**3.** Workplace ostracism condition^a^	—	—	.05	–.10	—				

**4.** Organizational dehumanization	4.74	1.54	.11	–.08	.75***	—			

**5.** Job satisfaction	3.77	1.99	–.05	.05	–.80***	–.77***	—		

**6.** Turnover intentions	4.68	2.06	.03	–.03	–.85***	.83***	–.88***	—	

**7.** Loyalty behaviors	3.59	1.82	–.09	.09	–.83***	–.80***	.88***	–.88***	—


*Note*: *N* = 199. ^a^ The experimental conditions were coded –1 = low workplace ostracism condition and +1 = high workplace ostracism condition *** *p* < .001.

### Results

Descriptive statistics and correlations between variables are provided in Table 2.

#### Measurement Model

Results of CFA indicated that the four-factor (i.e., organizational dehumanization, job satisfaction, turnover intentions, loyalty behaviors) model fitted the data well (χ^2^(203) = 421.46; RMSEA = .07; SRMR = .04; CFI =. 95; TLI = .95) and was superior to all more constrained models (see Table S3 in the online supplements).

#### Structural Model

**Figure 3:**
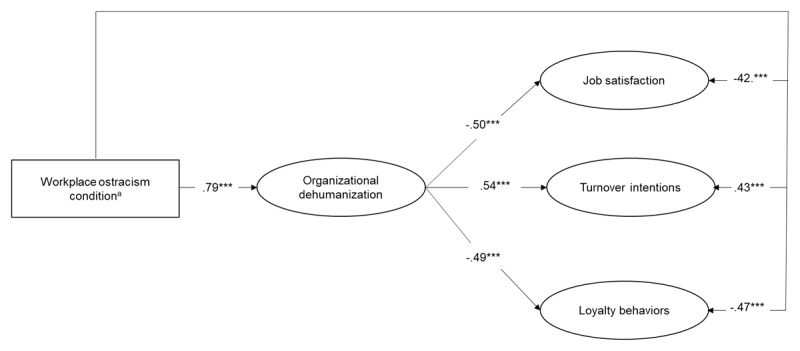
Standardized Coefficients for the Retained Structural Equation Model of Study 2. *Note*. *N* = 199. ^a^ Workplace ostracism condition was coded –1 for low workplace ostracism condition and +1 for high workplace ostracism condition. All constructs represented in ovals are latent variables. *** *p* < .001.

Again, SEM were performed to test the hypotheses. Specifically, we tested a model where the workplace ostracism condition (coded –1 for low and +1 for high workplace ostracism) relates to the final outcomes (i.e., job satisfaction, turnover intentions, loyalty behaviors), both directly and indirectly, through organizational dehumanization. Although the aforementioned model, including all possible direct paths between the condition and the outcomes, fitted the data well (χ^2^(221) = 460.27; RMSEA = .07; SRMR = .04; CFI = .95; TLI = .95), alternative models were tested removing direct paths one-by-one (see Table S4 in the online supplements). Results indicated that the model including all direct paths had a significantly better fit and was thus retained as the final model (Figure 3).

Results revealed that workplace ostracism had a positive impact on organizational dehumanization (γ = .79, *p* < .001), supporting Hypothesis 1. As displayed in Figure 3, workplace ostracism negatively impacted job satisfaction (γ = –.42, *p* < .001), positively affected turnover intentions (γ = .43, *p* < .001), and negatively impacted loyalty behaviors (γ = –.47, *p* < .001). In turn, organizational dehumanization was negatively related to job satisfaction (β = –.50, *p* < .001), positively related to turnover intentions (β = .54, *p* < .001), and negatively related to loyalty behaviors (β = –.49, *p* < .001). Latent bootstrap analyses (10000 bootstrap) also indicated that, the indirect effects of workplace ostracism on outcomes via organizational dehumanization were significant (job satisfaction = –.394, 95% CI [–.517, –.289]; turnover intentions = .426, 95% CI [.320, .538]; loyalty behaviors = –.388, 95% CI [–.508, –.284]), supporting Hypotheses 2b–d. Overall, organizational dehumanization partially mediated the relationships between workplace ostracism and the three outcomes.

## Study 3

### Method

#### Participants and Procedure

Similar to Study 1 and Study 2, participants who took part in this field study were recruited via Prolific Academic. They were paid £0.92 to complete the online questionnaire. To participate, they had to have an approval rate on the platform of at least 95%, be native English speakers, not be students nor self-employed, and not have taken part in Study 1 nor in Study 2. As 15 participants were excluded due to wrong answers to attentional questions, the final sample was composed of 423 participants (*M*_age_ = 39.94, *SD* = 9.95; 61.2% women, 38.8% men) who had an average organizational tenure of 8.41 years (*SD* = 7.19) and worked in the health and social care area (15.4%), the education domain (14.7%), and the retail and sales industry (9.9%). Most participants held a bachelor’s degree (40.9%) and worked full-time (78.3%). Moreover, the vast majority of them only worked remotely two days per week or less (73.8%).

#### Measures

Workplace ostracism (α = .95), organizational dehumanization (α = .96), depression (α = .85), job satisfaction (α = .94), turnover intentions (α = .95), and loyalty behaviors (α =.91) were measured using the same scales as in Study 1. Employees’ thwarted need to belong was assessed using the four items from Chen et al. (2015). Similar to Busque-Carrier et al. (2022), the items were contextualized to the work context by adding the label “At work” at the beginning of each item (e.g., “At work, I feel the relationships I have are just superficial”; α = .86). Previous research provided evidence for the psychometric properties of this scale (Busque-Carrier et al., 2022; Chen et al., 2015). Items were rated using a 7-point Likert scale ranging from “1” (strongly disagree) to “7” (strongly agree). Full scales are provided in the online supplements.

**Control Variables**. Again, we relied on scholars’ recommendations to deal with sociodemographic control variables (Aguinis et al., 2017; Becker 2005). As shown in Table 3, some sociodemographic variables are correlated with the dependent variables of the model. As their inclusion in the analyses did not change the main interpretations of the findings, the results reported here were free from any demographic variables for parsimony reasons (Becker, 2005). Similar to Study 1, abusive supervision was included as a control variable and was assessed with the same scale (α = .95).

**Table 3:** Descriptive Statistics and Correlations among Variables for Study 3.


VARIABLE	*M*	*SD*	1	2	3	4	5	6	7	8	9	10	11	12	13	14	15	16	17	18

**1.** *Gender*	–	–	–																	

**2.** *Age*	39.94	9.95	–.06	–																

**3.** Organizational sector	–	–	.17***	.05	–															

**4.** *Type of contract*	–	–	.29***	–.00	.11*	–														

**5.** *Hierarchical status*	–	–	.11*	–.14**	.15**	.20***	–													

**6.** *Organizational size*	–	–	–.02	–.05	.12**	–.05	.01	–												

**7.** *Organizational tenure (years)*	8.41	7.19	–.07	.43***	.02	.05	–.14**	.11*	–											

**8.** *Tenure–coworkers (years)*	5.00	4.09	.01	.32***	–.02	.12*	–.14**	–.08	.54***	–										

**9.** *Tenure–supervisor (years)*	4.13	4.09	–.09	.30***	–.07	.04	–.17***	.20***	.39***	.58***	–									

**10.** *Teleworking days per week*	1.45	1.72	–.17***	.01	–.05	–.26***	–.12*	.15**	–.03	–.10*	–.12*	–								

**11. Abusive supervision**	1.57	1.01	–.06	–.05	–.03	.04	.05	–.06	–.08	–.07	–.02	–.16**	–							

**12.** Workplace ostracism	1.92	1.08	–.00	–.08	–.05	.04	.08	.04	–.08	–.07	–.03	–.11*	.51***	–						

**13.** Thwarted need to belong	2.37	1.24	–.05	–.01	–.04	.02	.06	.03	–.08	–.11*	–.06	–.08	.46***	.67***	–					

**14.** Organizational dehumanization	3.94	1.53	.03	–.06	–.05	.05	.12*	.20***	–.04	–.11*	–.11*	–.18***	.35***	.34***	.44***	–				

**15.** Depression	3.12	1.48	.16***	–.11**	.08	.10*	.14**	.07	–.08	–.09	–.09	–.13**	.36***	.42***	.49***	.43***	–			

**16.** Job satisfaction	4.88	1.59	.05	.03	.02	–.03	–.13**	.02	.00	.08	.04	.18***	–.45***	–.39***	–.46***	–.61***	–.42***	–		

**17.** Turnover intentions	3.44	2.02	–.01	–.15**	–.03	.05	.13**	.01	–.13**	–.15**	–.16**	–.18***	.38***	.37***	.47***	.64***	.46***	–.75***	–	

**18.** Loyalty behaviors	4.60	1.43	.12**	.10	.02	–.02	–.26***	–.04	.00	.06	.08	.13**	–.34***	–.25***	–.33***	–.57***	–.26***	.70***	–.59***	–


*Note*: *N* = 423 (excepted for Type of contract *N* = 422). Gender was coded –1 = male and +1 = female. Organizational sector was coded –1 = private sector and +1 = public sector. Type of contract was coded 1 = full-time, 2 = 4/5 time, 3 = 3/4 time, and 4 = half-time. Hierarchical status was coded 1 = executive, 2 = supervisor management, 3 = middle manager, 4 = employee, and 5 = laborer. Organizational size was coded from 1 = less than 10 people to 9 = more than 10000 people. Abusive supervision is in bold because it was included as a control variable. The hypotheses were tested with and without the sociodemographic variables in italic as control variables but these were not included in the final model for parsimony reasons. * *p* < .05. ** *p* < .01. *** *p* < .001.

### Results

Descriptive statistics and correlations among variables are displayed in Table 3.

#### Measurement Model

Results of CFA (Mplus 8.5, MLR estimator) showed that the eight-factor (i.e., workplace ostracism, abusive supervision, thwarted need to belong, organizational dehumanization, depression, job satisfaction, turnover intentions, and loyalty behaviors) model fitted the data well (χ^2^(961) = 2029.04; RMSEA = .05; SRMR = .05; CFI = .92; TLI = .92) and was superior to all more constrained models (see Table S5 in the online supplements).

#### Structural Model

Again, SEM were performed to test the hypotheses. Specifically, we tested a model where workplace ostracism relates to the final outcomes (i.e., depression, job satisfaction, turnover intentions, loyalty behaviors), both directly and indirectly, through thwarted need to belong and then organizational dehumanization, while controlling for abusive supervision. Although the aforementioned model, including all possible direct paths between variables, fitted the data well (χ^2^(961) = 2029.04; RMSEA = .05; SRMR = .05; CFI = .92; TLI = .92), alternative models were tested removing these direct paths one-by-one (see Table S6 in the online supplements). Results revealed that an alternative model keeping only direct paths between (1) workplace ostracism and job satisfaction, (2) thwarted need to belong and depression, and turnover intentions as well as between (3) abusive supervision and job satisfaction, and loyalty behaviors was equivalent to the model including all direct paths (χ^2^(969) = 2041.39; RMSEA = .05; SRMR = .06; CFI = .92; TLI = .92). For the sake of parsimony, this alternative model was retained as the final model.

**Figure 4:**
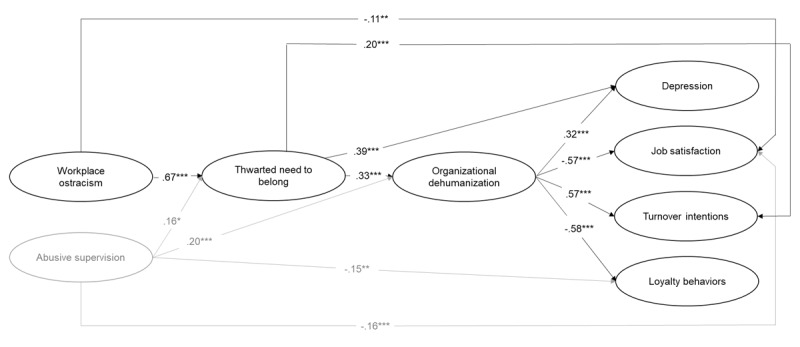
Standardized Coefficients for the Retained Structural Equation Model of Study 3. *Note*. *N* = 423. Abusive supervision was included as a control variable. All constructs represented in the figure are latent variables. * *p* < .05. ** *p* < .01. *** *p* < .001.

As shown in Figure 4, results indicated that workplace ostracism was positively related to thwarted need to belong (γ = .67, *p* < .001), which in turn was positively linked to organizational dehumanization (β = .33, *p* < .001). Ultimately, organizational dehumanization was positively related to depression (β = .32, *p* < .001), negatively related to job satisfaction (β = –.57, *p* < .001), positively related to turnover intentions (β = .57, *p* < .001), and negatively related to loyalty behaviors (β = –.58, *p* < .001), when controlling for abusive supervision. Furthermore, workplace ostracism was negatively related to job satisfaction (β = –.11, *p* < .01). Thwarted need to belong was also positively and directly associated with depression (β = .39, *p* < .001) and turnover intentions (β = .20, *p* < .001). Finally, latent bootstrap analyses (10000 bootstrap) revealed that, when abusive supervision is controlled for, the indirect effect of workplace ostracism on organizational dehumanization through employees’ thwarted need to belong was significant (indirect effect = .22, 95% CI [.144, .310]), supporting Hypothesis 3. Specifically, employees’ thwarted need to belong fully mediated the relationship between workplace ostracism and organizational dehumanization.

The hypothesized model (see Figure 1) suggests that workplace ostracism relates to employees’ well-being, attitudes, and behaviors via employees’ thwarted need to belong first, and then via organizational dehumanization. Therefore, complementary latent bootstrap analyses were conducted to examine this sequential double mediation. The indirect effects of workplace ostracism via thwarted need to belong and organizational dehumanization on outcomes were significant (depression = .07, 95% CI [.042, .113]); job satisfaction = –.13, 95% CI [–.184, –.081]); turnover intentions = .13, 95% CI [.081, .180]); loyalty behaviors = –.13, 95% CI [–.187, –.081]).

## Discussion

The primary aim of this research was to examine for the first time the positive relationship between workplace ostracism and organizational dehumanization as well as whether it subsequently relates to employees’ well-being (i.e., increased depression), attitudes (i.e., decreased job satisfaction) and behaviors (i.e., increased turnover intentions, decreased loyalty behaviors). Additionally, this research aimed to explore one underlying mechanism of the workplace ostracism-organizational dehumanization relationship, namely employees’ thwarted need to belong.

First, results of Study 1 and 3 provided evidence for the positive relationship between workplace ostracism and organizational dehumanization. Workplace ostracism was positively related to employees’ organizational dehumanization perceptions when controlling for another interpersonal mistreatment (i.e., abusive supervision) that was found to affect organizational dehumanization in prior studies (Caesens et al., 2019). These findings suggest that the absence of social attention at work (i.e., workplace ostracism), stemming from the supervisor or coworkers, relates to organizational dehumanization beyond perceived negative attention from one’s supervisor. Furthermore, a second experimental study using vignettes indicated that workplace ostracism is an antecedent of organizational dehumanization, thereby extending current knowledge of its interpersonal predictors (Stinglhamber et al., 2021). This highlights that the interpersonal predictors of organizational dehumanization are not limited to abusive supervision or leader-member exchange (Brison et al., 2022; Caesens et al., 2019). Rather, it shows that an interpersonal mistreatment that is commonly appraised as “mundane and innocuous given its indirect nature” (O’Reilly et al., 2015, p.775) can positively influence organizational dehumanization perceptions.

Second, across three studies, workplace ostracism was found to relate to poorer employees’ well-being (i.e., increased depression), attitudes (i.e., decreased job satisfaction), and behaviors (i.e., increased turnover intentions, decreased loyalty behaviors) through organizational dehumanization. Additionally, workplace ostracism was directly related to employees’ depression (Study 1), job satisfaction (Study 2, Study 3), turnover intentions (Study 2), and loyalty behaviors (Study 2), indicating that organizational dehumanization only partially mediates these relationships. Consistent with prior research (e.g., Howard et al., 2020), these findings further highlighted that workplace ostracism may directly affect victims’ well-being, attitudes, and work behaviors. However, some direct links did not replicate across studies (e.g., link between workplace ostracism and depression). This may be due to the specific context in which the study was conducted (e.g., COVID-19 pandemic; Study 1), the research design that was different (e.g., experimental study based on vignettes; Study 2) or the introduction of an additional mediator (e.g., Study 3). Overall, these partial mediation effects suggest that complementary underlying mechanisms might be at stake in the workplace ostracism-outcomes relationships. For instance, prior research showed that negative self-perceptions (e.g., global self-esteem; Bedi, 2021) and emotional reactions (e.g., anxiety; Ferris et al., 2016) accounted for the relationships between workplace ostracism and employees’ poorer well-being, attitudes (Bedi, 2021) and work behaviors (Bedi, 2021; Ferris et al., 2016). Nonetheless, this research adds to the workplace ostracism literature as it sheds light on one underlying mechanism (i.e., organizational dehumanization) that explains why experiencing workplace ostracism might influence different categories of negative employee outcomes (i.e., well-being, attitudes, behaviors; Li et al., 2021). This responds to scholars’ call to go beyond the existing fragmented literature on workplace ostracism by proposing a more integrated overview of the phenomenon (Howard et al., 2020). It also contributes to the organizational dehumanization literature and its nomological network (e.g., Caesens et al., 2017) as it is the first to provide evidence that organizational dehumanization is positively associated with employees’ levels of depression and negatively associated with employees’ loyalty behaviors. Supporting scholars’ claim that the negative consequences of organizational dehumanization expand beyond the professional world (e.g., Lagios et al., 2023), this research empirically shows that organizational dehumanization is negatively related to employees’ overall mental health.

Finally, results of Study 3 indicated that employees’ thwarted need to belong fully mediates the relationship between workplace ostracism and organizational dehumanization. In this regard, this research responds to scholars’ call to examine the mechanisms through which the predictors of organizational dehumanization foster these perceptions among employees (i.e., thwarted need to belong) (Brison et al., 2022). This adds to the organizational dehumanization literature as it highlights how interpersonal mistreatment (e.g., workplace ostracism) relates to organizational dehumanization. Consistent with prior work from Demoulin et al. (2020), results showed that organizational dehumanization arises from the frustration of employees’ need to belong. However, it is also argued that employees’ basic needs thwarting is a consequence of organizational dehumanization (Lagios et al., 2022). Future studies should thus examine whether these conflicting perspectives may be reconciled notably in the idea that a vicious circle is at play (Brison et al., 2022). Specifically, future studies should be conducted in order to see whether organizational dehumanization may thwart employees’ needs which, in turn, increases organizational dehumanization perceptions.

### Limitations and Future Research

This research has several limitations. First, two of our three studies were cross-sectional, thereby preventing any inference regarding causality. Yet, pertaining to the workplace ostracism-organizational dehumanization relationship, in Study 2, we relied on an experimental design to address this issue. Nevertheless, future studies relying on a two-factor experimental design should be conducted, as it is recommended by scholars to test mediation models experimentally (Pirlott & MacKinnon, 2016). Specifically, prior to measuring the dependent variables, participants would be randomly assigned to a high versus a low condition of the independent variable and to a high versus a low condition of the mediator (Pirlott & MacKinnon, 2016). A first experiment manipulating both workplace ostracism and organizational dehumanization, while measuring the dependent variables (i.e., job satisfaction, turnover intentions, loyalty behaviors) should be conducted. A second experiment manipulating both workplace ostracism and employees’ need to belong, while measuring organizational dehumanization could also be performed.

Second, although we argue that blame attributions (e.g., directed at the organization) might play a role in explaining the detrimental consequences of workplace ostracism, the extent to which victims blame a specific entity (e.g., the organization, the perpetrator, themselves) for their exclusion was not measured. Future experimental studies should thus be conducted to examine these attributional processes as possible explaining mechanisms underlying the relationships between workplace ostracism and outcomes. Concretely, after recalling one ostracizing event experienced within their current workplace, victims might indicate to what extent they attribute this to the organization or to alternative sources (e.g., perpetrator, themselves).

Third, this research relies on the workplace ostracism scale developed by Ferris et al. (2008). Although widely used, this scale does not precise who the ostracism stems from (i.e., supervisor, coworkers or the team), thereby being agnostic to power differentials between the perpetrators of ostracism (Ferris et al., 2008). Yet, since treatment from the supervisor, as compared to treatment from coworkers, has stronger relationships with organizational perceptions (Ng & Sorensen, 2008), it cannot be ruled out that the relationship that was highlighted between workplace ostracism and organizational dehumanization is driven by ostracism from the supervisor. Accordingly, in line with scholars’ call for more studies that explicitly contrast supervisor and coworker ostracism (e.g., Jahanzeb et al., 2018), future research should examine whether coworker ostracism affects organizational dehumanization and ultimately work-related outcomes beyond supervisor ostracism.

Finally, since this research relies on self-reported measures, the results may have been affected by common method variance (CMV; Podsakoff et al., 2003). However, drawing on Podsakoff’s et al. (2003) recommendations, several precautions were taken to limit this threat. For instance, confidentiality and anonymity of the participants’ responses were guaranteed and they were informed that there were no right or wrong answers to the questions. Additionally, we used validated scales that were separated in the questionnaire and a Harman’s single-factor test indicated that a one-factor solution fits the data poorly. That being said, scholars recently claimed that “the probability of significant distortion of estimates because of CMV is very limited” (Bozionelos & Simmering, 2022, p.194) and that self-reported measures should be favored when investigating employees’ subjective perceptions, like in the present study (Cruz, 2022).

### Practical Implications

This research holds several implications for practitioners. First and foremost, these findings underline the importance for organizations to minimize workplace ostracism. Because workplace ostracism is often a response to interpersonal conflict, organizations should pay particular attention to conflict management (Quade et al., 2017). For instance, offering mindfulness training is thought to improve employees’ conflict management skills (Scott & Duffy, 2015). Specifically, mindfulness training is argued to prevent ostracizing events by decreasing employees’ tendency to give in impulsive reactions, and by providing them with enhanced empathy and emotional regulation skills (Scott & Duffy, 2015). Additionally, managers are encouraged to reward employees’ ability to handle interpersonal conflict during their yearly evaluation (Scott & Duffy, 2015). Second, organizations should create an inclusive work environment by promoting cooperative objectives within their teams, rather than competitive goals (Wu et al., 2015). In the same vein, holding formal and informal gatherings to foster social interactions and interpersonal understanding is thought to be an effective way to mitigate workplace ostracism (Jahanzeb et al., 2020).

On the other hand, because organizational dehumanization was found to relate to deleterious consequences for both employees and the organization (i.e., increased depression, decreased job satisfaction, increased turnover intentions, and decreased loyalty behaviors), these perceptions should be reduced among employees. Concretely, in order to lessen organizational dehumanization, managers could improve employees’ sense of autonomy within their jobs (Demoulin et al., 2020) such as by providing flexible working arrangements. Additionally, leaders should strive to develop high-quality relationships with their followers as supervisor-subordinates interactions are predictors of subordinates’ organizational dehumanization perceptions (e.g., Stinglhamber et al., 2021). Finally, providing employees with a work environment that fulfills their basic psychological needs (Brison et al., 2022) by, for instance, prioritizing individual and customizable workspaces (Taskin et al., 2019) or by ensuring appropriate levels of noise and light (Stinglhamber et al., 2022).

## Conclusion

Overall, this research constitutes the first empirical evidence that being excluded or ignored at work by other organizational members (i.e., supervisor, coworkers) is associated with victims’ thwarted need to belong, which relates to their perceptions that the organization treats them as interchangeable tools. In turn, these perceptions are linked to employees’ poorer well-being (i.e., increased depression), work attitudes (i.e., decreased job satisfaction), and behaviors (i.e., increased turnover intentions, decreased loyalty behaviors).

## Data Accessibility Statement

Databases are available from the corresponding author upon request.

## Additional File

The additional file for this article can be found as follows:

10.5334/pb.1215.s1 Online Supplements. Measurement scales, Supplementary tables, and Experimental material.

## Footnotes

Hypothesis 2a was not tested in Study 2 because vignettes require participants to imagine themselves experiencing a situation rather than genuinely experiencing it, and are therefore inappropriate to assess emotional outcomes (e.g., depression) (Collett & Childs, 2011).
