# Supplementary material for: The Relationship Between Workplace Ostracism and Organizational Dehumanization: The Role of Need to Belong and its Outcomes
Source: Psychol Belg. 2023 Nov 8;63(1):120–37. doi: 10.5334/pb.1215 (PMC10637290; doi:10.5334/pb.1215)
Supplement: Online Supplements. — Measurement scales, Supplementary tables, and Experimental material. [file pb-63-1-1215-s1.pdf]

Measurement scales

<u>Workplace ostracism (Ferris et al., 2008)</u>
Please indicate the degree to which you agree with the following statements.
(1=strongly disagree; 2=disagree; 3=slightly disagree; 4=not agree, nor disagree; 5=slightly agree; 6=agree; 7=strongly agree)
Others ignored you at work
Others left the area when you entered
Your greetings have gone unanswered at work
You involuntarily sat alone in a crowded lunchroom at work
Others avoided you at work
You noticed others would not look at you at work
Others at work shut you out of the conversation
Others refused to talk to you at work
Others at work treated you as if you weren't there
Others at work did not invite you or ask you if you wanted anything when they went out for a coffee break

<u>Abusive supervision (Mitchell & Ambrose, 2007)</u>
Please indicate the degree to which you agree with the following statements.
(1=strongly disagree; 2=disagree; 3=slightly disagree; 4=not agree, nor disagree; 5=slightly agree; 6=agree; 7=strongly agree)
My supervisor ridicules me
My supervisor tells me my thoughts or feelings are stupid
My supervisor puts me down in front of others
My supervisor makes negative comments about me to others
My supervisor tells me I am incompetent

<u>Employees' thwarted need to belong (adapted from Chen et al., 2015 following Busque-Carrier's et al., 2022 procedure)</u>
Please indicate the degree to which you agree with the following statements.
(1=strongly disagree; 2=disagree; 3=slightly disagree; 4=not agree, nor disagree; 5=slightly agree; 6=agree; 7=strongly agree)
At work, I feel excluded from the group I want to belong to[1]
At work, I feel that people who are important to me are cold and distant towards me
At work, I have the impression that people I spend time with dislike me
At work, I feel the relationships I have are just superficial

[1] The original items from Chen et al. (2015) do not start with "at work". This contextualization is based on the work by Busque-Carrier et al. (2022).

Organizational dehumanization (Caesens et al., 2017)
Please indicate the degree to which you agree with the following statements.
(1=strongly disagree; 2=disagree; 3=slightly disagree; 4=not agree, nor disagree; 5=slightly agree; 6=agree; 7=strongly agree)
My organization makes me feel that one worker is easily as good as any other
My organization would not hesitate to replace me if it enables the company to make more profit
If my job could be done by a machine or a robot, my organization would not hesitate to replace me by this new technology
My organization considers me as a tool to use for its own ends
My organization considers me as a tool devoted to its own success
My organization makes me feel that my only importance is my performance at work
My organization is only interested in me when it needs me
The only thing that counts for my organization is what I can contribute to it
My organization treats me as if I were a robot
My organization considers me as a number
My organization treats me as if I were an object

Depression (Bohannon et al., 2003)
Please indicate the degree to which you agree with the following statements.
(1=strongly disagree; 2=disagree; 3=slightly disagree; 4=not agree, nor disagree; 5=slightly agree; 6=agree; 7=strongly agree)
I felt depressed
My sleep was restless
I felt lonely
I had crying spells
I could not get 'going'

Job satisfaction (Eisenberger et al., 1997)
Please indicate the degree to which you agree with the following statements.
(1=strongly disagree; 2=disagree; 3=slightly disagree; 4=not agree, nor disagree; 5=slightly agree; 6=agree; 7=strongly agree)
If a good friend of mine told me that he/she was interested working in a job like mine I would strongly recommend it
All in all, I'm very satisfied with my current job
In general, my job measures up to the sort of job I wanted when I took it
Knowing what I know now, if I had to decide all over again whether to take my job, I would

Turnover intentions (Jaros, 1997)
Please indicate the degree to which you agree with the following statements.
(1=strongly disagree; 2=disagree; 3=slightly disagree; 4=not agree, nor disagree; 5=slightly agree; 6=agree; 7=strongly agree)
I often think about quitting this organization
I intend to leave my organization in a near future
I intend to search for a position with another employer within the next year

Loyalty behaviors (adapted from Van Dyne et al., 1994)
Please indicate the degree to which you agree with the following statements.
(1=strongly disagree; 2=disagree; 3=slightly disagree; 4=not agree, nor disagree; 5=slightly agree; 6=agree; 7=strongly agree)
I represent my organization favorably to outsiders
I **go**[2] out of way to defend my organization against outside threats
I **tell**[3] outsiders this is a good place to work
I **defend**[4] my organization when employees criticize it

Supplementary Table S1.

[2] Original item: "I don't go out of way to defend my organization against outside threats"
[3] Original item: "I don't tell outsiders this is a good place to work"
[4] Original item: "I don't defend my organization when employees criticize it"

Model	χ^2	*df*	RMSEA	SRMR	CFI	TLI	SCF	$\Delta\chi^2_{SB}$	Δ_{df}
1. Seven-factor model	1279.24	798	.05	.04	.94	.94	1.15	---	---
2. Six-factor model (JS-LOY = 1 factor)	1441.99	804	.06	.05	.92	.92	1.15	162.75***	6
3. Six-factor model (TI-DEP = 1 factor)	1576.38	804	.06	.06	.91	.90	1.15	297.14***	6
4. Six-factor model (JS-TI = 1 factor)	1608.19	804	.06	.05	.90	.90	1.15	328.95***	6
5. Six-factor model (JS-DEP = 1 factor)	1652.53	804	.06	.08	.90	.89	1.15	373.29***	6
6. Six-factor model (TI-LOY = 1 factor)	1725.13	804	.07	.06	.89	.88	1.14	6718.77***	6
7. Six-factor model (DEP-LOY = 1 factor)	1757.95	804	.07	.09	.89	.88	1.15	478.71***	6
8. Six-factor model (WO-AS = 1 factor)	1933.62	804	.07	.06	.87	.86	1.16	309.99***	6
9. Six-factor model (AS-OD = 1 factor)	2246.98	804	.08	.12	.83	.82	1.17	302.31***	6
10. Six-factor model (WO-OD = 1 factor)	3184.49	804	.11	.15	.72	.70	1.18	442.28***	6
11. Five-factor model (WO-AS = 1 factor and JS-LOY = 1 factor)	2089.58	809	.08	.07	.85	.84	1.16	505.34***	11
12. Four-factor model (DEP-JS-TI-LOY = 1 factor)	2152.25	813	.08	.09	.84	.83	1.15	873.01***	15
13. Three-factor model (WO-AS = 1 factor and DEP-JS-TI-LOY = 1 factor)	2784.41	816	.10	.10	.77	.75	1.16	1096.96***	18
14. Two-factor model (WO-AS-OD = 1 factor and DEP-JS-TI-LOY = 1 factor)	4813.10	818	.14	.17	.52	.50	1.19	1527.80***	20
15. One-factor model	5609.78	819	.15	.17	.43	.40	1.20	1696.97***	21

Fit indices of measurement models for Study 1

Note. *N* = 256. RMSEA = root mean square error of approximation; SRMR = standardized root mean square residual; CFI = comparative fit index; TLI = Tucker-Lewis index; SCF =s caling correction factor; $\Delta\chi^2$ SB = strictly positive Satorra-Bentler chi-square difference test; AS = abusive supervision; WO = workplace ostracism; OD = organizational dehumanization; DEP = depression; JS = job satisfaction; TI = turnover intentions; LOY = loyalty behaviors.

***$p < .001$

Supplementary Table S2.

Model	χ2	*df*	RMSEA	SRMR	CFI	TLI	SCF	Δχ2 SB	Δ*df*
Hypothesized including all direct paths	1279.242	798	.05	.04	.94	.94	1.1464		
Alternative 1 (hypothesized - path between AS and LOY)	1279.696	799	.05	.04	.94	.94	1.1467	0.6424	1
Alternative 2 (alternative 1 - path between AS and TI)	1293.196	800	.05	.05	.94	.94	1.1465	15.4270***	1
Alternative 3 (alternative 1 - path between AS and JS)	1288.750	800	.05	.05	.94	.94	1.1468	8.5685**	1
Alternative 4 (alternative 1 - path between AS and DEP)	1285.413	800	.05	.05	.94	.94	1.1466	6.0252**	1
Alternative 5 (alternative 1 - path between WO and LOY)	1278.839	800	.05	.05	.94	.94	1.1475	0.0225	1
Alternative 6 (alternative 5 - path between WO and TI)	1279.062	801	.05	.05	.94	.94	1.1475	0.223	1
Alternative 7 (alternative 6 - path between WO and JS)	1279.022	802	.05	.05	.94	.94	1.1475	0.04	1
Alternative 8 (alternative 7 - path between WO and DEP)	1284.528	803	.05	.05	.94	.94	1.1474	5.7999*	1

Fit indices of structural models for Study 1

Note. N = 256. RMSEA = root mean square error of approximation; SRMR = standardized root mean square residual; CFI = comparative fit index; TLI = Tucker-Lewis index; SCF = scaling correction factor; $\Delta\chi^2$ SB = strictly positive Satorra-Bentler chi-square difference test; AS = abusive supervision; WO = workplace ostracism; DEP = depression; JS = job satisfaction; TI = turnover intentions; LOY = loyalty behaviors.

$^{*}p < .05$. $^{**}p < .01$. $^{***}p < .001$.

Vignettes of Study 2

"In this survey, you will be asked to imagine that you have been a Web Developer for 2 years at JPK Digit, a software company that is currently employing 257 workers. As a web developer, your main job responsibilities are related to designing websites and programming IT tools for JPK Digit's clients. You have always met JPK Digit's expectations in terms of work performance. On the next page, you will find a description of a typical working day at JPK Digit, from your arrival in the morning to your return home at the end of the day. This description is representative of your general experience at JPK Digit. Please read the story carefully and try to imagine that you are the employee in this story. Then please answer the following questions from the perspective of this employee."

High workplace ostracism condition

"Please read the story carefully and try to imagine that you are the employee in this story. Then please answer the following questions from the perspective of this employee.

9:00 am. You arrive at JPK Digit and walk into the open space. Several coworkers from your direct department are already working in front of their laptops. You greet them but get no answer. They barely look at you. You take a seat at a desk, turn on your laptop and start working on your main tasks.

10:30 am. Some coworkers arrived later than you but none of them came and sit next to you in the open space. You take a short break. Hoping to take your mind off the hard work you delivered this morning, you ask two colleagues working at another table whether they are having a good day. As your question remains unanswered, you get back to work.

1:00 pm. At lunchtime, you go to the JPK Digit self-service restaurant, get a meal and sit down at one of the many tables. The lunchroom gradually fills up but nobody joins you at your table. You quickly eat your meal and get back to work.

3:00 pm. You have an important meeting with your colleagues and your direct supervisor. During the meeting, an innovative idea for the future of JPK Digit is being debated. You raise your point to make a constructive suggestion but no one really seems to care. At the end of the meeting, you take some time to pack up your files and you then join your colleagues who were already standing. Once close to them, they leave the room and go to the coffee machine, leaving you behind although you just joined them.

5:30 pm. You leave the office to go back home."

Low workplace ostracism condition

"Please read the story carefully and try to imagine that you are the employee in this story. Then please answer the following questions from the perspective of this employee.

9:00 am. You arrive at JPK Digit and walk into the open space. Several coworkers from your direct department are already working in front of their laptops. You greet them. They look at you and they greet you back. You take a seat at a desk, turn on your laptop and start working on your main tasks.

10:30 am. Some coworkers arrived later than you and two of them came and sit next to you in the open space. You take a short break. Hoping to take your mind off the hard work you delivered this morning, you ask these two colleagues whether they are having a good day. They answer your question and you start talking together for a few minutes. Then, you get back to work.

1:00 pm. At lunchtime, you go to the JPK Digit self-service restaurant, get a meal and sit down at one of the many tables. The lunchroom gradually fills up and some coworkers from your direct department come and sit at your table. You quickly eat your meal and get back to work.

3:00 pm. You have an important meeting with your colleagues and your direct supervisor. During the meeting, an innovative idea for the future of JPK Digit is being debated. You raise your point and make a constructive suggestion, which sparked the interest of the others. At the end of the meeting, you take some time to pack up your files and you then join your colleagues who were already standing, waiting for you. At this point, a colleague leaves the room to go to the coffee machine. He suggests that you and the others follow him to share a coffee.

5:30 pm. You leave the office to go back home."

Manipulation check of Study 2 (multiple-choice question)

High workplace ostracism condition

Regarding the description of the day that you were asked to read at the beginning of the survey, which statement is true?

A. Your direct supervisor has entrusted you with the creation of a website for JPK Digit's most important client
B. Your colleagues did not pay attention to your suggestion during the meeting and they shared a coffee without you after the meeting
C. You have been told that you were going on a business trip next month, which really upsets you

Low workplace ostracism condition

Regarding the description of the day that you were asked to read at the beginning of the survey, which statement is true?

A. Your direct supervisor has entrusted you with the creation of a website for JPK Digit's most important client
B. Your suggestion during the meeting raised the interest of your colleagues and you shared a coffee with them after the meeting
C. You have been told that you were going on a business trip next month, which really upsets you

Model	χ^2	*df*	RMSEA	SRMR	CFI	TLI	SCF	$\Delta\chi^2_{SB}$	Δ_{df}
1. Four-factor model	421.463	203	.07	.04	.95	.95	1.1386	--	--
2. Three-factor model (JS-TI = 1 factor)	624.928	206	.10	.04	.91	.90	1.1752	69.70***	3
3. Three-factor model (JS-LOY = 1 factor)	570.637	206	.09	.04	.92	.91	1.1681	58.10***	3
3. Three-factor model (TI-LOY = 1 factor)	618.608	206	.10	.04	.91	.90	1.1692	75.13***	3
3. Two-factor model (JS-TI-LOY = 1 factor)	741.035	208	.11	.05	.87	.87	1.2010	109.82***	5
5. One-factor model	1194.849	209	.15	.06	.79	.77	1.2600	191.09***	6

Supplementary Table S3.

Fit indices of measurement models for Study 2

Note. *N* = 199. RMSEA = root mean square error of approximation; SRMR = standardized root mean square residual; CFI = comparative fit index; TLI = Tucker-Lewis index; SCF =s caling correction factor; $\Delta\chi^2$ SB = strictly positive Satorra-Bentler chi-square difference test; JS = job satisfaction; TI = turnover intentions; LOY = loyalty behaviors.

***$p < .001$

Supplementary Table S4.

Model	$\chi 2$	*df*	RMSEA	SRMR	CFI	TLI	SCF	$\Delta\chi 2$ SB	Δ*df*
Hypothesized including all direct paths	460.268	221	.07	.04	.95	.95	1.1295		
Alternative 1 (hypothesized - path between condition and JS)	492.708	222	.08	.05	.95	.94	1.1378	13.704***	1
Alternative 2 (hypothesized - path between condition and TI)	513.163	222	.08	.05	.94	.93	1.1315	38.622***	1
Alternative 3 (hypothesized - path between condition and LOY)	511.616	222	.08	.05	.94	.94	1.1351	25.651***	1

Fit indices of structural models for Study 2

Note. *N* = 199. RMSEA = root mean square error of approximation; SRMR = standardized root mean square residual; CFI = comparative fit index; TLI = Tucker-Lewis index; SCF = scaling correction factor; $\Delta\chi^2$ SB = strictly positive Satorra-Bentler chi-square difference test; JS = job satisfaction; TI = turnover intentions; LOY = loyalty behaviors.

$^{***}p < .001$.

Supplementary Table S5.

Model	χ^2	*df*	RMSEA	SRMR	CFI	TLI	SCF	$\Delta\chi^2_{SB}$	Δ_{df}
1. Eight-factor model	2029.04	961	.05	.05	.92	.92	1.25	---	---
2. Seven-factor model (WO-BEL = 1 factor)	2355.50	968	.06	.06	.90	.89	1.26	163.94***	7
3. Seven-factor (JS-LOY = 1 factor)	2368.22	968	.06	.06	.90	.89	1.25	339.19***	7
4. Seven-factor model (JS-TI = 1 factor)	2428.73	968	.06	.05	.89	.89	1.26	198.99***	7
3. Seven-factor model (TI-DEP = 1 factor)	2560.39	968	.06	.07	.88	.87	1.25	531.36***	7
5. Seven-factor model (JS-DEP = 1 factor)	2580.59	968	.06	.08	.88	.88	1.25	551.56***	7
7. Seven-factor model (DEP-LOY = 1 factor)	2705.35	968	.07	.09	.87	.87	1.25	557.33***	7
6. Seven-factor model (TI-LOY = 1 factor)	3170.91	968	.08	.08	.84	.83	1.26	743.37***	7
8. Seven-factor model (WO-AS = 1 factor)	3329.59	968	.08	.08	.82	.82	1.28	363.05***	7
9. Seven-factor model (AS-OD = 1 factor)	3603.79	968	.08	.11	.81	.80	1.28	404.01***	7
10. Seven-factor model (WO-OD = 1 factor)	4477.91	968	.09	.15	.75	.73	1.28	606.61***	7
11. Six-factor model (WO-BEL = 1 factor and JS-LOY = 1 factor)	2692.99	974	.07	.07	.87	.87	1.26	460.41***	13
12. Five-factor model (DEP-JS-TI-LOY = 1 factor)	3327.22	979	.08	.08	.83	.82	1.26	505.34***	18
13. Four-factor model (WO-BEL = 1 factor and DEP-JS-TI-LOY = 1 factor)	3637.41	983	.08	.09	.81	.80	1.27	1010.55***	22
14. Three-factor model (WO-BEL-OD = 1 factor and DEP-JS-TI-LOY = 1 factor)	6234.02	986	.11	.15	.62	.60	1.29	1347.09***	25
16. Two-factor model (WO-BEL-OD-AS = 1 factor and DEP-JS-TI-LOY = 1 factor)	7783.68	988	.13	.15	.51	.48	1.27	3913.10***	27
17. One-factor model	8419.73	989	.13	.15	.46	.44	1.32	2304.30***	28

Fit of measurement models for Study 3

Note. *N* = 423. RMSEA = root mean square error of approximation; SRMR = standardized root mean square residual; CFI = comparative fit index; TLI = Tucker-Lewis index; SCF = scaling correction factor; $\Delta\chi^2$ SB = strictly positive Satorra-Bentler chi-square difference test; AS = abusive supervision; WO = workplace ostracism; BEL = thwarted need to belong; OD = organizational dehumanization; DEP = depression; JS = job satisfaction; TI = turnover intentions; LOY = loyalty behaviors.

***$p < .001$.

Supplementary Table S6.

Model	χ2	*df*	RMSEA	SRMR	CFI	TLI	SCF	Δχ2 SB	Δ*df*
Hypothesized including all direct paths	2029.036	961	.05	.05	.92	.92	1.2517		
Alternative 1 (hypothesized - path between BEL and LOY)	2029.287	962	.05	.05	.92	.92	1.2515	0.865	1
Alternative 2 (alternative 1 - path between BEL and TI)	2035.717	963	.05	.05	.92	.92	1.2513	7.2150**	1
Alternative 3 (alternative 1 - path between BEL and JS)	2031.897	963	.05	.05	.92	.92	1.2515	2.61	1
Alternative 4 (alternative 3 - path between BEL and DEP)	2040.719	964	.05	.05	.92	.92	1.2511	11.8079**	1
Alternative 5 (alternative 3 - path between WO and OD)	2032.371	964	.05	.05	.92	.92	1.2512	0.0171	1
Alternative 6 (alternative 5 - path between AS and DEP)	2034.182	965	.05	.05	.92	.92	1.2510	1.7568	1
Alternative 7 (alternative 6 - path between AS and JS)	2050.532	966	.05	.06	.92	.92	1.2509	17.5405***	1
Alternative 8 (alternative 6 - path between AS and TI)	2037.325	966	.05	.05	.92	.92	1.2510	3.143	1
Alternative 9 (alternative 8 - path between AS and LOY)	2045.434	967	.05	.06	.92	.92	1.2508	9.2051**	1
Alternative 10 (alternative 8 - path between WO and DEP)	2040.473	967	.05	.06	.92	.92	1.2509	3.2350	1
Alternative 11 (alternative 10 - path between WO and JS)	2046.837	968	.05	.06	.92	.92	1.2508	6.7204*	1
Alternative 12 (alternative 10 - path between WO and TI)	2041.492	968	.05	.06	.92	.92	1.2507	0.8194	1
Alternative 13 (alternative 12 - path between WO and LOY)	2041.386	969	.05	.06	.92	.92	1.2508	0.0531	1

Fit indices of structural models for Study 3

Note. $N = 423$. RMSEA = root mean square error of approximation; SRMR = standardized root mean square residual; CFI = comparative fit index; TLI = Tucker-Lewis index; SCF = scaling correction factor; $\Delta\chi^2$ SB = strictly positive Satorra-Bentler chi-square difference test; BEL = thwarted need to belong; AS = abusive supervision; WO = workplace ostracism; OD = organizational dehumanization; DEP = depression; JS = job satisfaction; TI = turnover intentions; LOY = loyalty behaviors.

*$p < .05$. **$p < .01$. ***$p < .001$.
